# Bitter taste receptor T2R38 is expressed on skin-infiltrating lymphocytes and regulates lymphocyte migration

**DOI:** 10.1038/s41598-022-15999-6

**Published:** 2022-07-11

**Authors:** Moe Sakakibara, Hayakazu Sumida, Keisuke Yanagida, Sosuke Miyasato, Motonao Nakamura, Shinichi Sato

**Affiliations:** 1 Department of Dermatology, Faculty of Medicine, The University of Tokyo, 7-3-1, Hongo, Bunkyo-ku, Tokyo, 113-8655 Japan; 2 Department of Lipid Signaling, National Center for Global Health and Medicine, Shinjuku-ku, Tokyo, Japan; 3 Department of Bioscience, Graduate School of Life Science, Okayama University of Science, Okayama, Japan

**Keywords:** Chemokines, Lymphocytes, Cell migration, Skin diseases

## Abstract

Bitter taste receptors (T2Rs) are G protein-coupled receptors involved in the perception of bitter taste on the tongue. In humans, T2Rs have been found in several sites outside the oral cavity. Although T2R38 has been reported to be expressed on peripheral lymphocytes, it is poorly understood whether T2R38 plays immunological roles in inflammatory skin diseases such as atopic dermatitis (AD). Then, we first confirmed that T2R38 gene expression was higher in lesional skin of AD subjects than healthy controls. Furthermore, skin T2R38 expression levels were correlated with serum thymus and activation-regulated chemokine and IgE levels in AD patients. In lesional skin of AD, section staining revealed that CD3^+^ T cells in the dermis were T2R38 positive. In addition, flow cytometry analysis showed T2R38 expression in skin T cells. Migration assays using T2R38-transduced Jurkat T cell leukemia cells revealed that T2R38 agonists exerted a dose-dependent migration inhibitory effect. Moreover, skin tissue extracts, as well as supernatants of cultured HaCaT keratinocytes, caused T2R38-dependent migration inhibition, indicating that there should be an endogenous ligand for T2R38 in the skin epidermis. These findings implicate T2R38 as a migratory inhibitory receptor on the skin-infiltrating lymphocytes and as a therapeutic target for allergic/inflammatory skin diseases.

## Introduction

Bitter taste receptors (T2Rs) are G protein-coupled receptors (GPCRs) involved in bitter taste perception on the tongue^1^. Although 25 different T2Rs are known to be expressed in humans, T2Rs have recently been discovered in several regions outside the human oral cavity^2–4^. In particular, T2R38 has been found to be expressed in epithelial cells of the respiratory tract, colon, placenta, and peripheral neutrophils^5–8^. And most interestingly, Hoai T et al. have concluded that human T2R38 is expressed in resting and activated lymphocytes, suggesting the relevance of extra-gustatory T2R38 expression for human immunity^9^. Exploring knowledge about T2R38 function in human lymphocytes is essential to gain new insights into the potential role of T2R38 in the adaptive immune response.

In the field of dermatology, a variety of inflammatory skin diseases exist. In skin lesions, various immune cells, including lymphocytes, infiltrate the dermis from the early stage of the disease. For example, atopic dermatitis (AD) is the most common chronic inflammatory skin disease, characterized by sensitive, dry skin and eczematous lesions that are usually intensely itchy^10^. AD has a significant impact on the patient's quality of life. Accumulating evidence indicates that skin-infiltrating T cells, in particular T helper 2 (Th2) T cells, play a pivotal role during the initiation and maintenance of AD^11,12^. Hence, it is essential to understand the mechanisms regulating lymphocyte recruitment to the skin. Furthermore, understanding the mechanisms by which these immune cells infiltrate the skin and epidermis may lead to therapies that suppress skin inflammation from an early stage in various skin diseases. In addition, if disease- or tissue-specific molecules and mechanisms can be elucidated, it may lead to more specific treatment strategies. It has been known that C–C chemokine receptor type4 (CCR4) and C–C chemokine receptor type10 (CCR10) are important GPCRs that positively regulate the migration of lymphocytes (mainly T cells) homing into the skin, and they are relatively selectively expressed on skin lymphocytes^13^. However, analyses using deficient mice have shown that homing in the skin cannot be explained solely by existing molecules such as CCR4 and CCR10, thus the existence of other molecules that positively or negatively regulate homing has been suggested^14,15^.

Considering that most of the reported molecules involved in lymphocyte infiltration in the skin are GPCRs, we hypothesize that T2R38 may regulate the trafficking of distinct leukocyte subsets into skin tissues. We first confirmed the T2R38 expression on skin-infiltrating CD3^+^ lymphocytes in AD by immunostaining and flow cytometry. Next, in this study, we used a human acute T cell leukemia cell line Jurkat and planned to reveal the biological functions of T2R38 in lymphocytes. Using a combination of approaches, which included gene transfection and migration assays, we examined the role of T2R38 on Jurkat cell migration using T2R38 agonists, phenylthiocarbamide (PTC) and 6-n-propylthiouracil (RPOP) treatment. Furthermore, the migration assays were performed using skin tissue extracts and cell supernatants to test for the presence of endogenous ligands for T2R38.

## Results

### T2R38 was expressed on skin-infiltrating lymphocytes in AD

**Figure 1:**
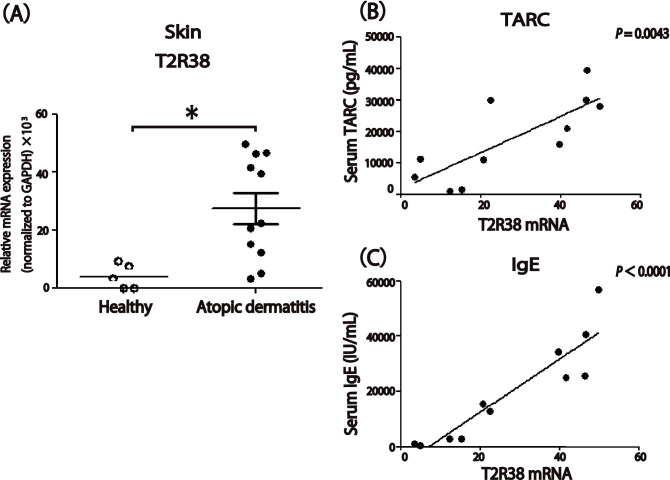
T2R38 expression in atopic dermatitis (AD) skin, and correlation between T2R38 expression levels in lesional skin and serum thymus and activation-regulated chemokine (TARC) or IgE levels in AD patients. (**A**) mRNA expression levels of T2R38 in healthy or AD skin. Skin tissue samples were obtained from 11 patients with AD and five healthy individuals. (**B**,**C**) Correlation of T2R38 expression levels in lesional skin and serum TARC (**B**) or IgE (**C**) levels in patients with AD. Skin tissue samples were obtained from 11 patients with AD. Significant positive correlation with T2R38 expression levels: TARC (r = 0.7840; *P* = 0.0043), IgE (r = 0.9171; *P* < 0.0001). *P < 0.05 by Student’s *t*-test. The correlation between two groups, in which the data points were distribution-free, was analyzed using the Pearson rank correlation coefficient. We attached row data for GAPDH in PCR assays from skin samples as a supplemental figure (Supplementary Table 1).

Gene expression analysis was carried out using skin tissue samples obtained from 11 patients with AD and five healthy individuals to characterize the relative mRNA expression levels of T2R38. T2R38 gene expression in lesional skin of AD subjects was significantly higher than that of healthy controls (*P* < 0.05; Fig. 1A). Moreover, in AD patients, T2R38 expression levels were correlated with serum thymus and activation-regulated chemokine (TARC; r = 0.7840, *P* = 0.0043) and IgE levels (r = 0.9171, *P* < 0.0001), diagnostic markers for assessing the severity of AD (Fig. 1B,C). We attached row data for GAPDH in PCR assays from skin samples as a Supplementary Table 1.

**Figure 2:**
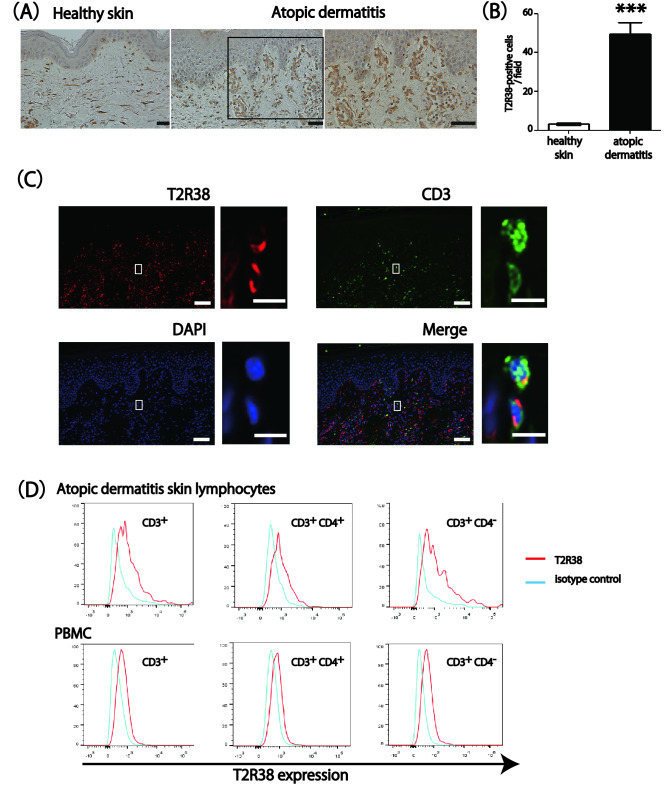
T2R38 expression on skin-infiltrating lymphocytes in atopic dermatitis (AD). (**A**) Representative images of immunohistochemical staining for T2R38 in lesional skin tissue from healthy control and AD. Bars = 100 μm. (**B**) Quantification of T2R38 positive cells/field in skin tissue. In the cell count of positive cells in immunohistochemical staining, stained cells were counted in 10 random grids under high-power fields. Skin tissue samples were obtained from four patients with AD and four healthy individuals. (**C**) Representative image of fluorescent double staining for CD3 and T2R38 in lesional skin tissue of AD. Bars = 100 μm. Enlarged images from rectangles in each panels are shown on the right. Bars = 20 μm. T2R38 = red, CD3 = green, DAPI = blue. (**D**) One representative of two independent experiments is shown. Flow cytometric analysis of T2R38 expression levels in skin-infiltrating lymphocytes in lesional skin AD and PBMC from AD patients. ***P < 0.001 by Student’s *t*-test.

Immunohistochemical analysis revealed that T2R38 was highly expressed in the infiltrating cells in the lesional skin of AD. In healthy skin, T2R38 was barely detected (*P* < 0.001; Fig. 2A,B). Sections stained with an isotype control antibody showed no detectable staining (data not shown). In addition, fluorescent staining for CD3 and T2R38 revealed the co-localization of these molecules (Fig. 2C). It should be noted that T2R38 seemed to stain cells other than lymphocytes by immunostaining (Fig. 2A). Consistently, some T2R38-positive cells were CD3 negative by fluorescent double staining (Fig. 2C). This suggested that T2R38 might be expressed on cells other than lymphocytes in the skin. Next, we performed flow cytometry to confirm that the T2R38-positive cells infiltrating the dermis in the above immunostaining were T-cell lymphocytes. We confirmed relative T2R38 expression on skin-infiltrating lymphocytes in lesional skin of AD and PBMC from AD patients by flow cytometry. Figure 2D depicts the results for T2R38 expression in both CD3^+^CD4^+^ and CD3^+^CD4^−^(CD8^+^) cells in AD skin and PBMC. Thus, T2R38 expression was observed in CD3^+^, CD3^+^CD4^+^, and CD3^+^CD8^+^ cells in the lesional skin of AD, along with a previous report showing T2R38 expression in lymphocytes of PBMC^9^. Based on the above immunostaining and flow cytometry results, we believe that T2R38 is expressed on skin lymphocytes.

### T2R38 transmitted a migration-inhibitory signal in response to PTC and PROP

**Figure 3:**
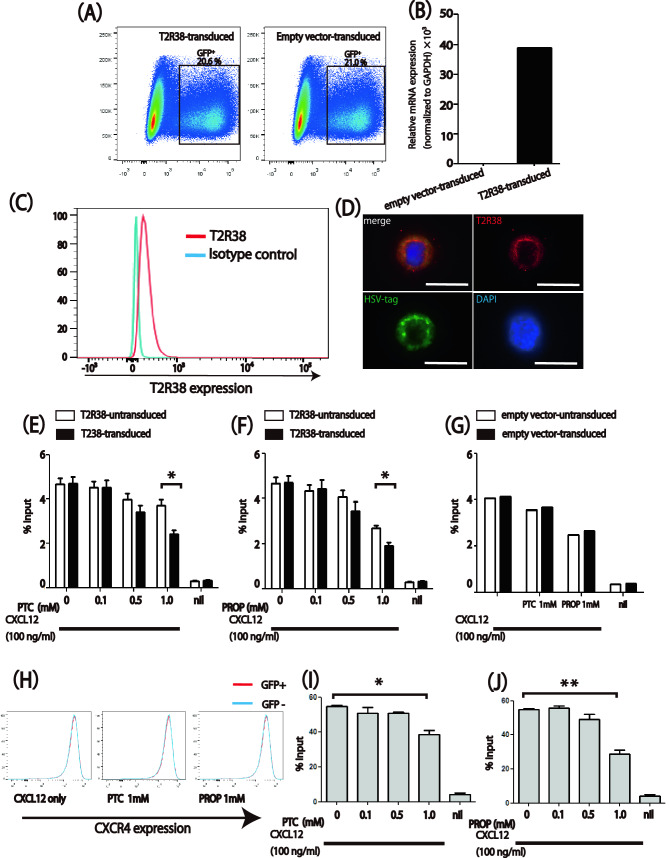
T2R38-dependent migration inhibition of Jurkat cells to recombinant human CXC chemokine ligand 12 (CXCL12) and the indicated amounts of phenylthiocarbamide (PTC) or 6-n-propylthiouracil (RPOP) in a transwell assay. (**A**,**B**) Confirmation of retroviral transfection of Jurkat cells using flow cytometry (**A**) and qPCR (**B**). qPCR was performed on the whole-cell lysate containing 80% untransduced cells and 20% transduced cells. (**C**) Flow cytometric analysis of T2R38 expression levels in T2R38 transduced Jurkat cells. (**D**) Cell fluorescence staining in T2R38-transfected cells. T2R38 = red, HSV tag = green, DAPI = blue. Bars = 10 μm. (**E**,**F**) Migration of T2R38 transduced Jurkat cells to CXCL12 and the indicated amounts of agonists PTC (**E**) or PROP (**F**). (**G**) Migration of empty vector-transduced Jurkat cells. (**H**) Flow cytometric analysis of CXCR4 expression levels in T2R38-transduced and untransduced cells. (**I**,**J**) Migration of human CD3^+^ CD4^+^ cells to CXCL12 and indicated amounts of T2R38 agonist PTC (**I**) or PROP (**J**). PTC and PROP were mixed with CXCL12 (100 ng/ml). The white bars indicate untransduced cells, and black bars indicate transduced cells. One representative of three independent experiments is shown. (**E**,**F**) *n* = 3; (**G**) *n* = 2; (**I**,**J**) *n* = 3 *P < 0.05 by Student’s *t*-test.

Since the expression of T2R38 was intrinsically low in Jurkat cells, we decided to analyze its function in transfection experiments. Jurkat cells were retrovirally transduced with retroviral vectors containing T2R38 or empty construct with GFP reporter. Transfection efficiency was about 20% for both T2R38 and empty vector by flow cytometry (Fig. 3A), and we also confirmed the increased expression of T2R38 in T2R38-transduced cells by qPCR (Fig. 3B). In addition, flow cytometry suggested the expression of T2R38 on the cell surface (Fig. 3C). Given that an HSV (herpes simplex virus) epitope tag was inserted into the C-terminus of the vector, we stained with a monoclonal antibody for the tag to examine the localization of the transduced T2R38. Furthermore, fluorescence microscopy using the anti-T2R38 antibody showed that transduced-T2R38 was expressed on the plasma membrane in transduced cells (Fig. 3D).

Using T2R38-transduced Jurkat cells in Transwell migration assays, we observed a dose-dependent migration-inhibitory effect of PTC and PROP in response to the chemoattractant CXCL12 (*P* < 0.05; Fig. 3E,F). No selective migration-inhibitory effect of PTC or PROP was observed on control Jurkat cells transduced with an empty vector (Fig. 3G). Incidentally, no difference in CXCR4 expression between T2R38-positive and negative cells was observed even under the PTC or PROP condition (Fig. 3H). As an approach to test the similar effects of the endogenously expressed T2R38 on the lymphocytes, we performed migration assays with isolated human PBMCs, and observed a dose-dependent migration inhibitory effect of PTC and PROP on CXCL12 (P < 0.05; Fig. 3I,J).

### Endogenous T2R38 ligand activity was found in skin extracts and HaCaT cell culture supernatants

**Figure 4:**
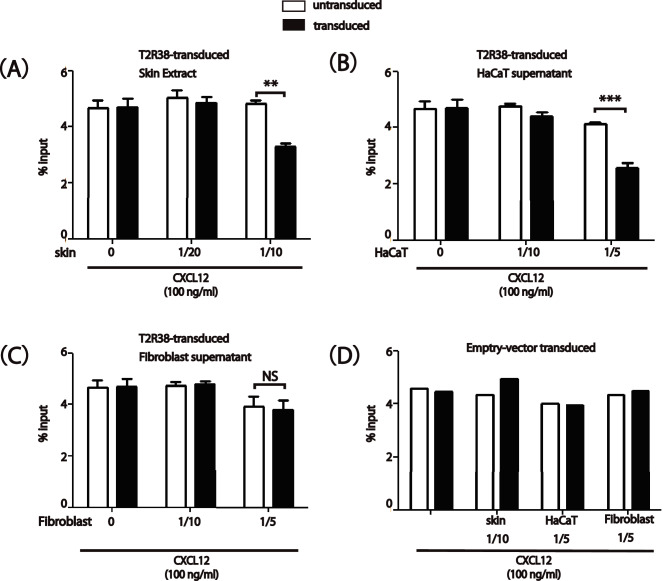
Endogenous T2R38 ligand activity in skin extract and cell culture supernatants from HaCaT cells but not from fibroblasts. (**A**–**C**) Migration of T2R38 transduced Jurkat cells to recombinant human CXC chemokine ligand 12 (CXCL12) and the indicated amounts of diluted skin extract (**A**), HaCaT cell culture supernatants (**B**), or fibroblast cell culture supernatants (**C**). (**D**) Migration of empty vector-transduced Jurkat cells. Skin extract and cell culture supernatants were mixed with CXCL12 (100 ng/ml). The white bars indicate untransduced cells, and black bars indicate transduced cells. One representative of three independent experiments is shown. (**A**–**C**) *n* = 3; (**D**) *n* = 2. ***P < 0.001, **P < 0.01 by Student’s *t*-test.

As an approach to testing for the presence of endogenous ligands for T2R38 in the skin, we used the migration inhibition technique as a bioassay, following the experiment with the agonists in Fig. 3. Skin tissue extracts caused T2R38-dependent migration inhibition (*P* < 0.01; Fig. 4A). Moreover, supernatants from cultured HaCaT cells contained T2R38 ligand activity using the bioassay (*P* < 0.001; Fig. 4B). Inhibition of migration of skin tissue extracts as well as supernatants of HaCaT cells was observed in a dose-dependent manner unless the extracts were extremely concentrated. We measured no T2R38-dependent inhibition by supernatants from human primary fibroblast (Fig. 4C). No selective migration-inhibitory effect was observed on control Jurkat cells transduced with an empty vector (Fig. 4D). These results suggest that the endogenous ligand for T2R38 may be produced by the skin, even by keratinocytes.

### Suppression of TNF-alpha production by PTC and PROP was not T2R38-dependent in Jurkat cells

**Figure 5:**
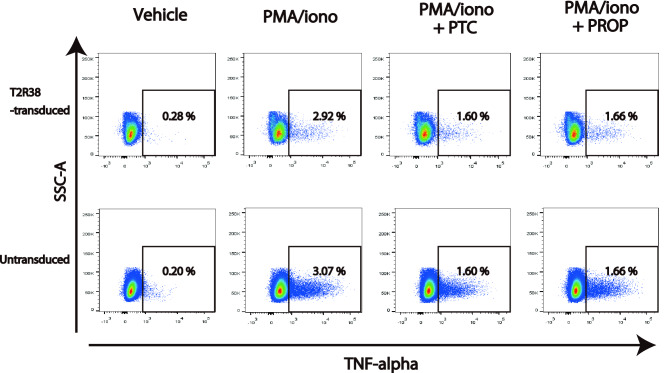
Comparable tumor necrosis factor (TNF)-alpha production of T2R38-transduced and T2R38-untransduced Jurkat cells to phenylthiocarbamide (PTC) and 6-n-propylthiouracil (RPOP). Jurkat cells were activated with ionomycin (1 μg/ml) and PMA (25 ng/ml) for 4 h w/wo the addition of PTC or PROP. Cells were fixed and permeabilized for flow cytometry. One representative of three independent experiments is shown.

A previous report has shown that T2R38 suppresses the potent proinflammatory cytokine TNF-alpha production in PBMC^9^. Since TNF-alpha production is also observed in Jurkat cells upon stimulation with PMA/ionomycin^16^, we then tested the impact of PTC and PROP on the TNF-alpha secretion using T2R38-transduced Jurkat cells. PTC and PROP reduced TNF-alpha secretion in Jurkat cells, but the extent of this reduction was similar in T2R38-transduced and T2R38-untransduced cells and was not considered to be T2R38-dependent (Fig. 5).

## Discussion

To the best of our knowledge, this is the first report suggesting that T2R38 is functionally expressed on skin-infiltrating lymphocytes. T2R38 was expressed in CD4^+^ and CD8^+^ T cells from lesional AD skin as well as PBMC. In addition, T2R38 expression levels in the skin were correlated with serum TARC and IgE levels in AD patients. We also revealed that T2R38 transmitted a migration-inhibitory signal in response to PTC and PROP in T2R38-transduced Jurkat cells. Of interest, there was a relative T2R38 ligand activity in skin extract and HaCaT cell culture supernatants. These results suggest that T2R38 is involved in lymphocyte migration in the skin.

In recent years, transcripts of multiple T2R genes have been detected in human skin biopsies^17,18^. A previous report has demonstrated that T2R14 is functionally expressed in epidermal keratinocytes^19^. However, little has been done to understand the function of T2Rs in the skin compared with other organs. A review has discussed that T2Rs recognize bacterial products that cause sinonasal infection^20^. The skin, as the external surface of a body, is frequently attacked by environmental factors, including pathogens. Immune cells act as sentinels and effector cells reside among the keratinocytes, fibroblasts, and endothelial cells that constitute skin. Given these, it is possible and reasonable that T2R38 is expressed on skin-infiltrating lymphocytes and plays a role in skin immunity.

We observed a correlation between T2R38 mRNA expression levels in skin lesions and serum TARC levels in patients with AD. Chemokine receptors, such as CCR4, have been shown to regulate the trafficking of distinct leukocyte subsets into peripheral tissues^21,22^. In particular, TARC (CCL17), a ligand for CCR4, is involved in lymphocyte-endothelial interactions during lymphocyte recruitment to cutaneous sites^14,23^. Here, T2R38 may inhibit TARC-mediated homing of T cells to the skin. To confirm the function of the T2R38 on lymphocytes, we examined the effects of the receptor on the agonist-induced chemotaxis. T2R38 is known to be activated by PTC and PROP, and previous reports have demonstrated the specificity of PTC and PROP for T2R38 compared to other T2Rs^24^. Consistently, in our T2R38-transduced Jurkat cells, T2R38 was shown to be activated specifically by the respective agonists compared with the empty vector. Importantly, the results from bioassay also suggested that T2R38 acted in an inhibitory manner on the migration of Jurkat cells. Our results agree with the previously reported role of T2R8 and T2R10 in abrogating the migration of neuroblastoma cells^25^. Notably, as well as these T2Rs, GPCRs other than chemokine receptors engage in multiple downstream pathways to modulate immune cell migration in barrier organs such as skin and mucosa^13,26,27^.

Endogenous ligands for T2Rs are poorly characterized, except a single study highlighting a steroid hormone, progesterone, activating mouse T2R110 and T2R114^28^. Interestingly, we confirmed migration inhibition in T2R38-transduced cells by skin tissue extract and HaCaT cell supernatant. Therefore, it is plausible that T2R38 activated by endogenous ligands in keratinocytes may play a role in skin immunity. Our results suggest that the endogenous ligand for T2R38 is located in the skin, and further analysis may identify the endogenous ligand for T2R38 in the future. And if the endogenous ligand for T2R38 could be identified, it could be administered subcutaneously as a target for anti-inflammatory drugs.

No T2R38-dependent inhibition of TNF-alpha release was detected upon PTC or PROP exposure in T2R38-transduced Jurkat cells. A previous study has shown that the addition of goitrin, an agonist for T2R38, inhibits TNF-alpha release in PBMC^9^. This discrepancy may be due to the difference in the sample: T2R38-transduced Jurkat cell was used in this study, while patient PBMC was used in the previous study. Furthermore, we did not use Goitrin in this experiment because goitrin was not a specific agonist for T2R38. This may also be attributed to differences in agonists. In our study, PROP and PTC, which showed T2R38-dependent migration inhibition in T2R38-transduced cells, affected the TNF-alpha production in a T2R38-independent manner. Although PTC and PROP exerted a T2R38-dependent inhibitory effect on migration in Jurkat cells, it remains possible that PTC and PROP also acted on other receptors in Jurkat cells to suppress cytokine production. This is a subject for future research.

Animal studies, including the ovalbumin-induced AD in mice^29^, are needed to elucidate the function of T2Rs in skin diseases. These studies will provide deeper insights into T2R roles in the pathophysiology of skin lymphocytes. However, a previous study has demonstrated that mouse orthologue Tas2r138 does not respond to PROP^28^. Furthermore, a recent study using the CRISPR/Cas9 gene-editing technique to delete three major T2Rs expressed in airway smooth muscle has shown that the T2R triple deletion does not impact the bronchodilation induced by T2R agonists^30^. We could not perform mouse experiments for these reasons, and mouse experiments are the most critical task for us in the future.

In summary, our data revealed the expression of T2R38 in human skin, suggesting that T2R38 may regulate T cell recruitment into inflamed skin. Given that T2R38 may be a regulator of skin immune homeostasis, the role of T2R38 in skin allergy and inflammatory diseases should be investigated in the future. Our findings hold the potential to pave the way for an appropriate and effective design for treating patients with inflammatory skin diseases. Moreover, these insights may be extrapolated to a broad spectrum of inflammatory and immune disorders other than skin diseases. They may aid in the rational design of novel anti-inflammatory treatments.

## Materials and methods

### Patients

This study was carried out by the ethical guidelines of the 1975 Declaration of Helsinki. It was approved by the Institutional Research Ethics Committee of the Faculty of Medicine of the University of Tokyo. Informed consent was obtained from the patients for the use of all samples. Blood was collected from volunteers after obtaining written informed consent. Skin samples were collected from 11 patients with AD and five healthy controls.

### Quantitative PCR (qPCR)

RNA was extracted using TRIzol (Thermo Fisher Scientific, Carlsbad, CA). TaqMan primers (FAM-MGB linked, Thermo Fisher Scientific) were used to assay for expression of human T2R38 (cat # Hs00604294_s1) and GAPDH (cat # Hs02786624_g1) on an Applied Biosystems real-time PCR machine (Thermo Fisher Scientific). We normalized T2R38 gene expression to GAPDH.

### Section staining

Tissue sections (5 μm thick) from formaldehyde-fixed and paraffin-embedded samples were de-waxed and rehydrated. In immunohistochemical staining, sections were stained with goat anti-rabbit T2R38 polyclonal IgG antibody (Sigma-Aldrich, Oakville, Canada) or goat anti-rabbit IgG control (Cell signaling, Danvers, MA) followed by ABC staining (Vector Laboratories, Burlingame, CA). Diaminobenzidine was used to visualize the staining, and counterstaining with Mayer’s hematoxylin was performed. In fluorescent staining, sections were stained with goat anti-rabbit T2R38 polyclonal IgG antibody (Sigma-Aldrich) and anti-mouse monoclonal CD3 (clone F7.2.38; Abcam), followed by second antibodies. A 1:200 dilution of Cy3-conjugated goat anti-rabbit-IgG (Jackson Immunoresearch, West Grove, PA), AF488-conjugated donkey anti-mouse-IgG (Jackson Immunoresearch), and 4′,6-diamidino-2-phenylindole (DAPI) were used to visualize the co-localization of CD3 and T2R38 signals. Regarding the specificity of the T2R38 antibody we used, there has been a report demonstrating the specificity of this antibody in HEK293T cells overexpressing T2R38^31^. Western blotting with anti-T2R38 antibody has also been performed using commercially available tongue tissue^32^. Moreover, pre-absorption of the T2R38 antibody with the corresponding antigenic peptides (T2R38 peptide) nearly totally abolished the signal with the anti-T2R38 antibody^32^.

### Immunocytochemistry

Jurkat cells were collected and smeared using Smear Gel (GenoStaff, Tokyo, Japan) according to manufacturer instructions and then fixed with 4% paraformaldehyde (PFA) in phosphate-buffered saline (PBS). After permeabilization with 0.2% Triton X-100 in PBS, being blocked in PBS containing 1% BSA, the slices were stained with goat anti-rabbit T2R38 polyclonal IgG antibody (Sigma-Aldrich) and anti-HSV-Tag antibody (Sigma-Aldrich; 69171), and incubated overnight at 4 °C. The antigen was detected with Cy3-conjugated goat anti-rabbit-IgG (Jackson Immunoresearch) and AF647-conjugated goat anti-mouse IgG (Jackson Immunoresearch), and nuclei were counterstained with DAPI solution (Dojindo Laboratories). Fluorescent images were taken with Zeiss Axio Observer (Carl Zeiss, Göttingen, Germany).

### Cell preparation and flow cytometry

Human peripheral blood mononuclear cells (PBMCs) were isolated from the fresh peripheral blood of volunteers. PBMC were separated from the blood by centrifugation on a Ficoll gradient (Sigma-Aldrich). Skin lymphocytes were isolated after digestion in RPMI 1640 supplemented with 500 U/ml collagenase type II (Worthington Biochemical Corporation, Lakewood, CA), 0.1 mg/ml deoxyribonuclease I (DNase I; Sigma-Aldrich), and 10% fetal calf serum (FCS) at 37 °C for 30 min. The following primary human antibodies labeled with fluorophore were used for flow cytometry: CD3-Phycoerythrin (PE; clone UCHT1), CD4-Phycoerythrin with Cyanin-7 (PE/Cy7; clone OKT4), and CXCR4-PE/Cy7 (clone 12G5) antibody from Biolegend (San Diego, CA). The primary antibody, goat anti-rabbit T2R38 polyclonal IgG antibody, goat anti-rabbit IgG control, and the fluorophore-labeled secondary antibodies, anti-rabbit-Alexa 488, were purchased from Abcam (Cambridge, United Kingdom). The fluorescence intensity of the cells was evaluated by flow cytometry (BD FACSVerse; BD Bioscience),

### Retroviral transduction

Human T cell leukemia cell line Jurkat cells (RIKEN Cell Bank, Tsukuba, Japan) were retrovirally transduced with MSCV2.2 retroviral vectors (Vector Builder, Chicago, IL) containing T2R38 with functional haplotype (PAV haplotype) or empty vector and an internal ribosome entry site (IRES)-Green Fluorescent Protein (GFP) reporter^33^. We inserted an HSV tag into the C-terminus. Briefly, the virus was produced using Platinum-A (Plat-A) cells (Cell Biolabs Inc, San Diego, CA), a potent retrovirus packaging cell line based on the 293 T cell line. Jurkat cells were spin-infected for 2 h with retroviral supernatant and cultured for migration assays.

### Migration assays and bioassays

Transwell migration assays were performed following a previously similar method^34^. Recombinant human CXC chemokine ligand 12 (CXCL12; Peprotech, Cranbury, NJ) was diluted to 100 ng/ml in the migration medium [RPMI plus 0.1% fatty acid-free bovine serum albumin (BSA) plus penicillin/streptomycin (P/S)]. PTC (Sigma-Aldrich) or PROP (Sigma-Aldrich) were diluted at varying concentrations in the CXCL12-containing migration medium. Then, 600 μl of these chemo-attractant mixtures was added into the bottom of the lower chamber in a 24-well tissue culture plate. 100 μl of the cell suspension was added to the top well of 5 μm Transwell inserts (Costar, Cambridge, MA) in a 24-well plate. To assess migration inhibition, the number of T2R38-GFP^+^ cells that migrated for each well was divided by the number of T2R38-GFP^+^ cells that were inputted into the upper chamber. In the migration assay with PBMCs, PBMCs migrating to the lower chamber were stained and analyzed by flow cytometry to confirm the number of cells of the CD3^+^CD4^+^ subset migrating to the lower chamber.

The bioassay for T2R38 ligand activity was performed as described previously^35^. Briefly, to test bioactivity production, fresh human skin tissue obtained from donors was mashed through a metal mesh and washed in a migration medium. The spontaneously immortalized human keratinocyte cell line HaCaT is often used as a model to study keratinocyte functions. The fibroblasts were cultured from primary human skin biopsy specimens. HaCaT and fibroblast cells were allowed to reach confluence, and the medium was then replaced with serum-free medium; incubated for 16–18 h, and tested in the bioassay. T2R38-or empty vector-transduced Jurkat cells were used, and the tissue extracts or cell supernatants were mixed with CXCL12, as indicated.

### Cytokine assay

Jurkat cells were activated with 1 μg/ml ionomycin (purity ≥ 98%) and 25 ng/ml phorbol 12-myristate 13-acetate (PMA; purity ≥ 98%) from Cayman Europe (Ann Arbor, MI) for 4 h w/wo the addition of PTC or PROP. Cells were fixed and permeabilized using BD Cytofix/Cytoperm™ Fixation/Permeabilization Kit (BD Biosciences, Heidelberg, Germany) according to the manufacturer’s protocol before staining with antibodies. The following primary human antibodies labeled with fluorophore were used for flow cytometry: tumor necrosis factor (TNF)-alpha-PE/Cy7 (clone Mab11) from Biolegend.

### Statistical analysis

Data were expressed as the mean ± SEM and analyzed using GraphPad Prism 6 software (GraphPad Software). Statistical significance was determined by a Student’s *t*-test. The correlation between two groups, in which the data points were distribution-free, was analyzed using the Pearson rank correlation coefficient. A value of *P* < 0.05 was considered statistically significant.

## Supplementary Information

Supplementary Table 1.

## Data Availability

The data that support the findings of this study are available from the corresponding author upon reasonable request.
