# Supplementary material for: Bitter taste receptor T2R38 is expressed on skin-infiltrating lymphocytes and regulates lymphocyte migration
Source: Sci Rep. 2022 Jul 11;12:11790. doi: 10.1038/s41598-022-15999-6 (PMC9276799; doi:10.1038/s41598-022-15999-6)
Supplement: Supplementary file 1 — Supplementary Table 1. [file 41598_2022_15999_MOESM1_ESM.pdf]

Supplementary information

Bitter taste receptor T2R38 is expressed on skin-infiltrating lymphocytes and regulates lymphocyte migration

Moe Sakakibara[1], Hayakazu Sumida[1*], Keisuke Yanagida[2], Sosuke Miyasato[3], Motonao Nakamura[3], Shinichi Sato[1]

Affiliations of the authors

[1]Department of Dermatology, Faculty of Medicine, The University of Tokyo, Bunkyo-Ku, Tokyo, Japan.

[2]Department of Lipid Signaling, National Center for Global Health and Medicine, Shinjuku-ku, Tokyo, Japan.

[3]Department of Bioscience, Graduate School of Life Science, Okayama University of Science, Okayama, Japan.

Corresponding author: Hayakazu Sumida, MD, PhD

Department of Dermatology, Faculty of Medicine, The University of Tokyo, 7-3-1, Hongo, Bunkyo-Ku, Tokyo, 113-8655, Japan.

E-mail: sumida-tky@umin.ac.jp

Tel.: +81 3 5800 8661

Fax: +81 3 3814 1503

Supplementary Table1

Row data for *GAPDH* in PCR assays from skin samples

sample name	Target	CT value
Healthy skin No.1	*GAPDH*	23.96975708
Healthy skin No.2	*GAPDH*	22.54921341
Healthy skin No.3	*GAPDH*	22.74416351
Healthy skin No.4	*GAPDH*	21.17991638
Healthy skin No.5	*GAPDH*	21.6879158
AD skin No.1	*GAPDH*	22.31202698
AD skin No.2	*GAPDH*	22.04991722
AD skin No.3	*GAPDH*	23.56932449
AD skin No.4	*GAPDH*	23.06023407
AD skin No.5	*GAPDH*	20.09181595
AD skin No.6	*GAPDH*	21.82665062
AD skin No.7	*GAPDH*	23.96928406
AD skin No.8	*GAPDH*	22.99201775
AD skin No.9	*GAPDH*	22.86565018
AD skin No.10	*GAPDH*	21.79933929
AD skin No.11	*GAPDH*	22.20566368

AD, Atopic dermatitis
